# Correlation Analysis of Anti-SARS-CoV-2 RBD IgG and Neutralizing Antibody against SARS-CoV-2 Omicron Variants after Vaccination

**DOI:** 10.3390/diagnostics12061315

**Published:** 2022-05-25

**Authors:** Nuchjira Takheaw, Chalerm Liwsrisakun, Warawut Chaiwong, Witida Laopajon, Supansa Pata, Juthamas Inchai, Pilaiporn Duangjit, Chaicharn Pothirat, Chaiwat Bumroongkit, Athavudh Deesomchok, Theerakorn Theerakittikul, Atikun Limsukon, Pattraporn Tajarernmuang, Nutchanok Niyatiwatchanchai, Konlawij Trongtrakul, Watchara Kasinrerk

**Affiliations:** 1 Division of Clinical Immunology, Department of Medical Technology, Faculty of Associated Medical Sciences, Chiang Mai University, Chiang Mai 50200, Thailand; nuchjira.t@cmu.ac.th (N.T.); witida.l@cmu.ac.th (W.L.); supansa.pata@cmu.ac.th (S.P.); 2 Biomedical Technology Research Center, National Center for Genetic Engineering and Biotechnology, National Science and Technology Development Agency at the Faculty of Associated Medical Sciences, Chiang Mai University, Chiang Mai 50200, Thailand; 3 Division of Pulmonary, Critical Care, and Allergy, Department of Internal Medicine, Faculty of Medicine, Chiang Mai University, Chiang Mai 50200, Thailand; chalerm.liw@cmu.ac.th (C.L.); warawut.chai@cmu.ac.th (W.C.); juthamas.i@cmu.ac.th (J.I.); pilaiporn.th@cmu.ac.th (P.D.); chaicharn.p@cmu.ac.th (C.P.); chaiwat.b@cmu.ac.th (C.B.); athavudh.d@cmu.ac.th (A.D.); theerakorn.t@cmu.ac.th (T.T.); atikun.limsukon@cmu.ac.th (A.L.); pattraporn.t@cmu.ac.th (P.T.); nutchanok.n@cmu.ac.th (N.N.); konlawij.tr@cmu.ac.th (K.T.)

**Keywords:** COVID-19, SARS-CoV-2, variants of concern, Omicron variant, COVID-19 vaccine

## Abstract

Various vaccines have been developed to control the COVID-19 pandemic, but the available vaccines were developed using ancestral SARS-CoV-2 wild-type (WT) strains. Commercial anti-SARS-CoV-2 receptor binding domain (RBD) antibody assays have been established and employed for validation of vaccine efficacy. However, these assays were developed before the SARS-CoV-2 variants of concern (VOCs) emerged. It is unclear whether anti-RBD IgG levels can predict immunity against VOCs. In this study, we determined the correlations between the levels of anti-RBD IgG and neutralizing antibodies (NAbs) against SARS-CoV-2 variants in vaccinated subjects. After vaccination, 100% of subjects showed an anti-RBD IgG response, whereas 82, 79, 30, 75, and 2% showed NAb responses against WT, Alpha, Beta, Delta, and Omicron variants, respectively. A high correlation was observed between anti-RBD IgG and NAbs against WT, Alpha, Beta, and Delta, but not so for the Omicron NAbs. Among subjects with high levels of anti-RBD IgG, 93, 93, 71, 93, and 0% of them had NAbs against WT, Alpha, Beta, Delta, and Omicron variants, respectively. These results indicate that anti-RBD IgG levels cannot be used as a predictor for the presence of NAbs against the globally dominant SARS-CoV-2 Omicron variant.

## 1. Introduction

Coronavirus diseases 2019 (COVID-19), caused by the SARS-CoV-2 virus, rapidly spread worldwide starting in early 2020. This pandemic has constituted a major threat to humans. To stop the pandemic, several COVID-19 vaccines were developed. Within one and a half years after the identification of SARS-CoV-2, almost 20 vaccines were authorized for emergency use worldwide [1,2]. All of these vaccines were developed using ancestral SARS-CoV-2 Wuhan strains. In that period, COVID-19 vaccines were demonstrated to induce humoral and cellular immunity to protect against SARS-CoV-2 infection, hospitalization, and death [1,3,4,5,6]. The presence of antibodies in vaccinated individuals was shown to correlate with protection from SARS-CoV-2 infection and symptomatic COVID-19 [7,8,9]. Thus, vaccination was considered the most important measure to control the COVID-19 pandemic.

However, during replication, SARS-CoV-2 changes its genome over time. Several variants have emerged from the wild-type (WT) strain, and some have been considered as variants of concern (VOCs) [10,11,12]. After the appearance of SARS-CoV-2 VOCs, particularly Delta and Omicron, there were reports of breakthrough infections despite full vaccination [12,13,14]. Currently, the Omicron variant has become the globally dominant variant [11]; there is no more ancestral WT virus, and the incidence of other VOCs is very low. The emergence of Omicron raised serious concerns due to the potential for immune escape from vaccine-induced antibodies [12,15,16,17]. This is because the available vaccines were established before the VOCs emerged, and the Omicron variant harbors a number of mutations in its S protein [12,18,19]. The Omicron mutation sites are the immunodominant targets for neutralizing antibodies (NAbs) elicited by COVID-19 vaccines [20,21].

Presently, serological tests are utilized to monitor the immune response after vaccination. Antibody assays for detecting anti-SARS-CoV-2 receptor binding domain (RBD) antibodies are available and suggested for use in validating vaccine efficacy. Anti-RBD IgG can be measured using SARS-CoV-2 RBD as an antigen. As these tests were developed before the VOCs emerged, the RBD antigens used in the assays are derived from the WT virus. Nevertheless, detection of anti-RBD IgG has been commonly employed to determine vaccine efficacy for SARS-CoV-2 variants. Yet, it is unclear whether anti-RBD IgG levels can predict the level of NAbs against SARS-CoV-2 VOCs, particularly Omicron. In this study, we compared the levels of anti-RBD IgG using SARS-CoV-2 IgG II Quant (Abbott) and surrogate NAbs against WT, Alpha, Beta, Delta, and Omicron variants using cPass SARS-CoV-2 neutralization antibody detection kits (GenScript) in unvaccinated and vaccinated persons. Our results suggest that the levels of anti-RBD IgG cannot be used to assume levels of NAbs against the Omicron variant.

## 2. Materials and Methods

### 2.1. Study Participants

This study enrolled 67 participants. Among them, 11 participants did not receive any vaccine, and 56 participants received either 2 doses of CoronaVac (Sinovac Biotech Co., Ltd., Beijing, China) or 1 dose of each of CoronaVac and ChAdOx-1 (AstraZeneca/University of Oxford, Oxford, UK). Blood samples were taken 4–12 weeks after the second vaccine dose, or upon enrollment for unvaccinated subjects. Plasma was separated and tested for levels of anti-RBD IgG or NAbs against WT, Alpha, Beta, Delta, and Omicron variants.

This study was approved by the Ethical Committee of the Faculty of Medicine, Chiang Mai University (IRB approval number: MED-2564-08247) and filed with the Clinical Trials Registry (study ID: TCTR20210822002). Before enrollment, written informed consent was obtained from all subjects.

### 2.2. Anti-Receptor Binding Domain (RBD) IgG Assay

Antibodies against the spike RBD of SARS-CoV-2 were quantitatively measured by the SARS-CoV-2 IgG II Quant chemiluminescence immunoassay using the ARCHITECT I System (Abbott Laboratories, Abbot Park, IL, USA), according to the manufacturer’s protocol. The sequence of the RBD antigen used in the test kit was taken from the WH human 1 coronavirus (GenBank accession number MN908947). The levels of anti-RBD IgG antibodies were presented in arbitrary units (AU/mL), and the analytical measurement range stated by the manufacturer is from 21 to 40,000 AU/mL. The obtained AU/mL values were then converted into WHO international standard concentrations (binding antibody units/mL, BAU/mL) following the equation provided by the manufacturer (BAU/mL = 0.142 × AU/mL). Antibody levels greater than or equal to the cut-off value of 7.1 BAU/mL (50 AU/mL) were defined as seropositive.

### 2.3. Neutralizing Antibody (NAb) Assay

Plasma specimens were determined for NAb against the SARS-CoV-2 WT, Alpha (B.1.1.7), Beta (B.1.351), Delta (B.1.617.2), and Omicron (B.1.1.529) variants by cPass SARS-CoV-2 neutralization antibody detection kit (GenScript, Piscataway, NJ, USA), according to the manufacturer’s protocol. Briefly, the kit contains the horseradish peroxidase (HRP) conjugated recombinant SARS-CoV-2 RBD fragment (HRP-RBD) of the WT strain and of the VOCs as listed: Alpha (Mutation sites at N501Y), Beta (Mutation sites at E484K, K417N and N501Y), Delta (mutation sites at L452R and T478K), and Omicron (mutation sites at G339D, S371L, S373P, S375F, K417N, N440K, G446S, S477N, T478K, E484A, Q493R, G496S, Q498R, N501Y, Y505H). The HRP-RBD of each strain was diluted 1:1000 with RBD dilution buffer. The plasma, positive, and negative controls were diluted to 1:9 using sample dilution buffer. The diluted samples and controls were incubated with the HRP-RBD solution at 1:1 ration for 30 min at 37 °C. One hundred microliters of each mixture was added into a human angiotensin-converting enzyme 2 (ACE2)-coated well and incubated for 15 min at 37 °C. Unbound proteins were removed by washing for 4 times. 3,3′,5,5′-Tetramethylbenzidine (TMB) substrate solution was added into each well and incubated for 15 min at room temperature. After the addition of the stop solution, the absorbance was measured at 450 nm using a microtiter plate reader. The percentage of inhibition of NAb was calculated as follows: (1 − (O.D. value of sample/average O.D. value of negative control from the corresponding strain)) × 100. According to the manufacturer (GenScript, Picataway, NJ, USA), the 30% inhibition was used as the cut-off, where % inhibition above the cut-off be considered as the NAb for SARS-CoV-2 was detected.

### 2.4. Statistical Analysis

Statistical analysis was performed using GraphPad Prism v9.2.0 (San Diego, CA, USA). Correlations between anti-RBD-IgG and NAbs against SARS-CoV-2 variants were determined using Spearman’s correlation coefficient analysis. The following parameters were used to justify the correlations: 0 < |r| < 0.3 = weak correlation; 0.3 < |r| < 0.7 = moderate correlation; |r| > 0.7 = strong correlation.

## 3. Results

### 3.1. Correlation of Anti-SARS-CoV-2 RBD IgG and Neutralizing Antibodies against SARS-CoV-2 Variants

**Figure 1:**
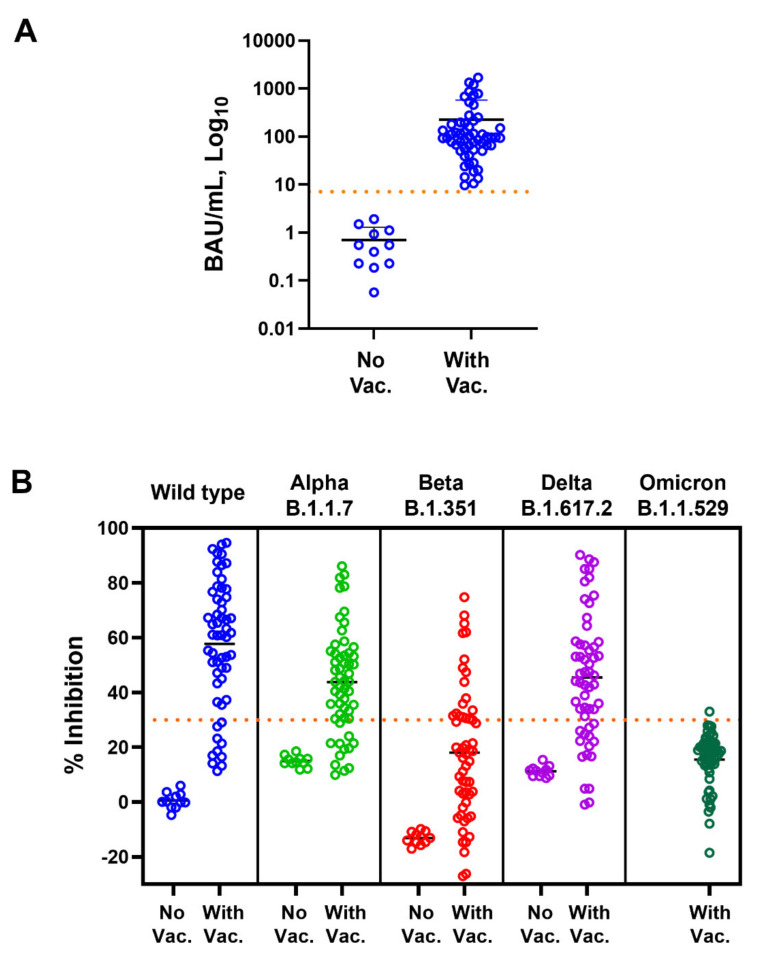
Anti-SARS-CoV-2 RBD IgG and neutralizing antibody responses in unvaccinated and vaccinated subjects. Levels of (**A**) anti-RBD IgG (BAU/mL) and (**B**) neutralizing antibody (% inhibition) against wild-type, Alpha, Beta, Delta, and Omicron variants of unvaccinated (No Vac.) and vaccinated (With Vac.) subjects are shown. Dot points represent individuals; mean ± SD are indicated; dotted horizontal lines represent cut-off values.

Among unvaccinated subjects, as expected, anti-SARS-CoV-2 RBD IgG was not detected (<7.1 BAU/mL cut-off) in any tested subject (Figure 1A). Concurrent with the anti-RBD IgG level, NAbs against WT, Alpha, Beta, and Delta variants were not detected (<30% inhibition cut-off) in unvaccinated persons (Figure 1B).

**Figure 2:**
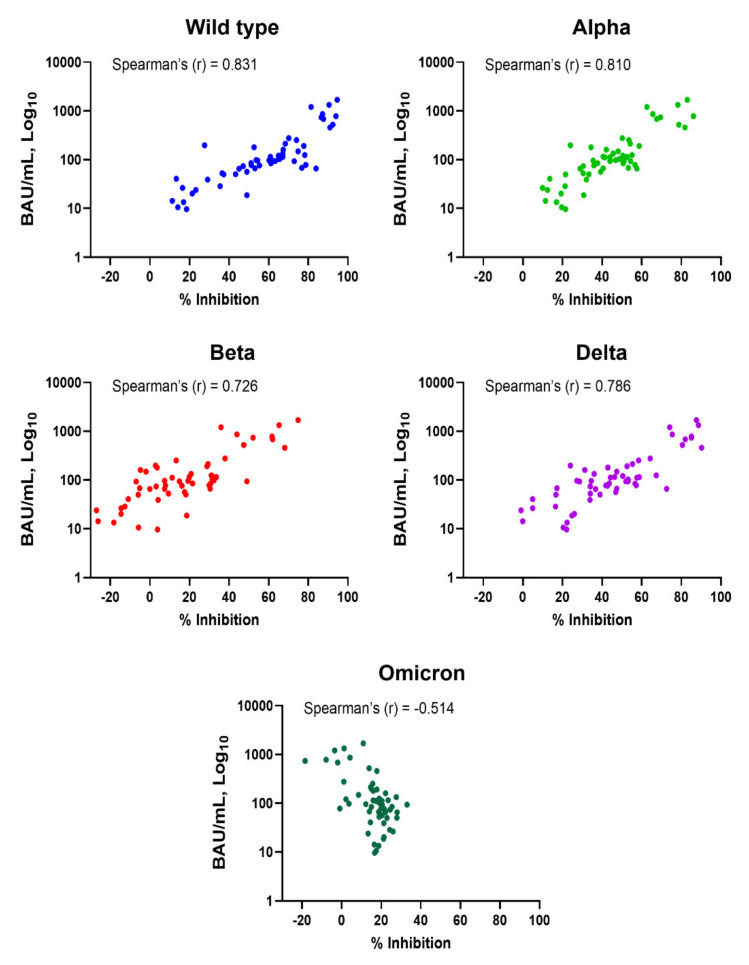
Correlation between levels of anti-SARS-CoV-2 RBD IgG and neutralizing antibodies in vaccinated subjects. Comparison between levels of anti-RBD IgG (BAU/mL) and neutralizing antibodies (% inhibition) against wild-type, Alpha, Beta, Delta, and Omicron variants is shown. Dot plots represent individuals. Spearman’s correlation coefficient (r) is indicated in each comparison.

Subjects who had been vaccinated with two doses of CoronaVac or one dose of CoronaVac and ChAdOx-1 had anti-RBD IgG levels above the cut-off (Figure 1A). NAbs against SARS-CoV-2 variants were also induced, but not in all subjects, and they varied depending on the tested variant (Figure 1B). Spearman’s correlation coefficients for anti-RBD IgG and Nabs against WT, Alpha, Beta, Delta, and Omicron variants were 0.831, 0.810, 0.726, 0.786, and −0.514, respectively (Figure 2 and Table 1). The results indicate that anti-RBD IgG levels did not correlate with Nab against Omicron; there was actually a negative correlation.

### 3.2. Vaccine Induced Anti-RBD IgG Response but Not Neutralizing Antibody against SARS-CoV-2 Omicron Variant

We further investigated the antibody response in vaccinated subjects. Among those who were vaccinated, 100% of tested subjects showed an anti-RBD IgG antibody response (Figure 1). However, in 82.14, 78.57, 30.36, 75.00, and 1.76% of subjects, NAbs were detected against WT, Alpha, Beta, Delta, and Omicron variants at levels above the cut-off value (Figure 1B).

**Figure 3:**
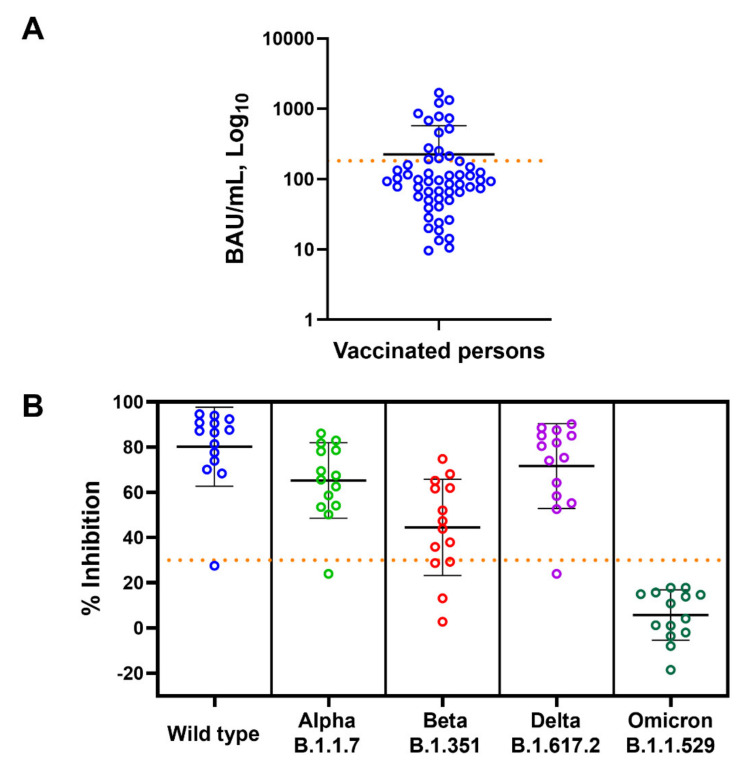
Anti-SARS-CoV-2 RBD IgG and neutralizing antibody levels of the vaccinated subjects. (**A**) Levels of anti-RBD IgG (BAU/mL) of all vaccinated subjected are shown as mean ± SD. Dotted horizontal line represents value of 182 BAU/mL used to identify subjects with high anti-RBD IgG titer. (**B**) Neutralizing antibody (% inhibition) against wild-type, Alpha, Beta, Delta, and Omicron variants in high-titer anti-RBD IgG subjects is shown as mean ± SD. Dotted horizontal line represents 30% inhibition cut-off values.

According to the US FDA guidelines for the therapeutic use of COVID-19 convalescent plasma [22], high-titer convalescent plasma is defined as having ≥182 BAU/mL anti-SARS-CoV-2 RBD IgG, as determined by Abbott ARCHITECT. We then used this value to identify high-titer COVID-19 samples (Figure 3A) and determined the presence of NAbs of SARS-CoV-2 variants in these subjects (N = 14). Among the subjects who had anti-RBD IgG of more than 182 BAU/mL, 93, 93, 71, 93, and 0% of them had NAbs against WT, Alpha, Beta, Delta, and Omicron variants, respectively (Figure 3B).

Altogether, these results indicate that in people inoculated with vaccine developed from the WT strain, measurement of anti-SARS-CoV-2 RBD IgG cannot be used to indicate the presence of NAbs against the Omicron variant.

## 4. Discussion

It is currently accepted that vaccination is an importance public health measure to control the COVID-19 pandemic. Several vaccines, with different platforms, were developed shortly after SARS-CoV-2 emerged in Wuhan, China. The spike (S) protein of SARS-CoV-2 is the targeted immunogen in most vaccine development [10]. This is because the RBD is the virus region that binds to host cell angiotensin-converting enzyme 2 (ACE2). The RBD is located in the S1 subunit of S protein, and binding of virus RBD and host cell ACE-2 initiates virus entry [23]. NAbs, antibodies that bind to RBD, can prevent the virus from entering host cells [10,24]. Thus, it is expected that vaccinated people will develop anti-SARS-CoV-2 S protein antibodies, along with anti-RBD antibodies and NAbs. The antibodies induced by vaccination are presumed to prevent SARS-CoV-2 infection.

Previous studies demonstrated that high titers of anti-RBD antibodies and NAbs were induced in vaccinated individuals [7,8,9]. A high correlation between RBD binding antibodies and NAbs was revealed [25]. Therefore, some health care settings and private health services use anti-RBD IgG levels to predict vaccine effectiveness in vaccinated individuals. However, the commercially available anti-RBD IgG assays were developed before the SARS-CoV-2 VOCs emerged; these assays were established based on the RBD of the ancestral WT strain as antigen. Nevertheless, soon after the pandemic with the ancestral virus, new variants of SARS-CoV-2 arose from the WT strain. Unfortunately, the mutations occurred at the RBD, which is the target site of NAbs [11,12,18,19]. Several VOCs of SARS-CoV-2 have been reported by WHO [10,12]. Each variant gradually became the globally dominant variant for a certain period of time. The occurrence of new variants was caused by breakthrough COVID-19 infections, despite full vaccination [13,14,26]. Evidence of evading vaccine-induced immunity to SARS-CoV-2 VOCs has been reported [27]. This raised the question of whether the levels of anti-RBD IgG as determined by commercially available assays can indicate the presence of Nabs against the currently circulating VOCs.

In the present report, we compared the levels of anti-RBD IgG, using Abbott’s SARS-CoV-2 IgG II Quant assay, and Nabs against WT, Alpha, Beta, Delta, and Omicron variants, using GenScript cPass SARS-CoV-2 neutralization antibody detection kits. In unvaccinated subjects, we confirmed that anti-RBD IgG and Nabs against WT, Alpha, Beta, and Delta were absent from their plasma. Although the NAbs against Omicron were not determined, we assumed that NAbs against the Omicron variant was also absent in unvaccinated subjects. After vaccination with two doses of CoronaVac or one dose of CoronaVac and ChAdOx-1, antibodies to RBD were induced in all subjects. On the other hand, NAbs were not induced in all subjects. The anti-RBD IgG levels had a strong correlation with NAbs against WT, Alpha, Beta, and Delta variants based on Spearman’s correlation coefficient analysis. However, there was no correlation between anti-RBD IgG and NAbs against Omicron. These results are in agreement with the knowledge that this variant contains several mutations in the RBD of the S protein. Actually, SARS-CoV-2 Omicron harbors many mutations in structural and nonstructural proteins. More than 32 mutations were found in the S protein, and 15 of these mutations reside in the RBD [12,18,19,28]. These mutations affect the binding of NAbs generated by vaccination [12,15,16,17]. Resistance to neutralization by convalescent serum of COVID-19 patients or people vaccinated against the Omicron strains has been documented [29]. It is worth mentioning that, in this study, the vaccinated subjects received two doses CoronaVac or received one dose of CoronaVac and one dose of ChAdOx-1. Our results indicate that even with the different immunogenicity of the vaccine used, there was no correlation between the levels of anti-RBD IgG and neutralizing antibodies against the Omicron variant.

We have also demonstrated that among vaccinated individuals with high anti-RBD IgG levels, none of them had detectable NAbs against Omicron. These vaccinated persons are therefore still at high risk of infection by the globally circulating SARS-CoV-2 Omicron variant. However, there are some limitations of our study. First, the blood collection was at 4–12 weeks after vaccination. The blood collecting time after vaccination may affect the levels of both anti-RBD IgG and NAbs. Second, the subjects who had subclinical SARS-CoV-2 infections were not identified and were not excluded. This might have affected the levels of detected antibodies.

## 5. Conclusions

Currently, commercial anti-RBD antibody assays are widely used in several countries, particularly by private health services, for monitoring vaccine-induced immunity. The results presented in this study indicate that the levels of anti-RBD IgG cannot be used to assume the levels of NAbs against Omicron variant and cannot be used as a predictor of the presence of SARS-CoV-2 protective immunity to Omicron. This has made it necessary to produce a new version of the assay that incorporates antigen derived from mutated RBD. This new assay, perhaps, could lead to a better correlation between anti-RBD antibodies and NAb levels.

## Figures and Tables

**Table 1:** Correlation between the levels of anti-RBD IgG and % inhibition of neutralizing antibodies against SARS-CoV-2 wild-type, Alpha, Beta, Delta, and Omicron variants (N = 56).

SARS-CoV-2 Variants	Spearman^®^(r)	*p*-Value
Wild-type	0.831	<0.001
Alpha	0.810	<0.001
Beta	0.726	<0.001
Delta	0.786	<0.001
Omicron	−0.514	<0.001

## Data Availability

The data used to support the findings of this study are included within the article.
